# Acute Response and Neuroprotective Role of Myo/Nog Cells Assessed in a Rat Model of Focal Brain Injury

**DOI:** 10.3389/fnins.2021.780707

**Published:** 2021-12-07

**Authors:** Sahlia Joseph-Pauline, Nathan Morrison, Michael Braccia, Alana Payne, Lindsay Gugerty, Jesse Mostoller, Paul Lecker, E-jine Tsai, Jessica Kim, Mark Martin, Rushil Brahmbhatt, Grzegorz Gorski, Jacquelyn Gerhart, Mindy George-Weinstein, Jonathan Stone, Sivaraman Purushothuman, Arturo Bravo-Nuevo

**Affiliations:** ^1^Philadelphia College of Osteopathic Medicine, Philadelphia, PA, United States; ^2^School of Medical Sciences, University of Sydney, Sydney, NSW, Australia; ^3^Discipline of Physiology, University of Sydney, Sydney, NSW, Australia; ^4^Brain and Mind Centre and Central Clinical School, University of Sydney, Sydney, NSW, Australia

**Keywords:** Myo/Nog cells, focal brain injury, rat brain, neuroprotection, BAI1

## Abstract

Focal brain injury in the form of a needlestick (NS) results in cell death and induces a self-protective response flanking the lesion. Myo/Nog cells are identified by their expression of bone morphogenetic protein inhibitor Noggin, brain-specific angiogenesis inhibitor 1 (BAI1) and the skeletal muscle specific transcription factor MyoD. Myo/Nog cells limit cell death in two forms of retinopathy. In this study, we examined the acute response of Myo/Nog cells to a NS lesion that extended from the rat posterior parietal cortex to the hippocampus. Myo/Nog cells were identified with antibodies to Noggin and BAI1. These cells were the primary source of both molecules in the uninjured and injured brain. One day after the NS, the normally small population of Myo/Nog cells expanded approximately eightfold within a 1 mm area surrounding the lesion. Myo/Nog cells were reduced by approximately 50% along the lesion with an injection of the BAI1 monoclonal antibody and complement. The number of dying cells, identified by terminal deoxynucleotidyl transferase-mediated dUTP-biotin nick end labeling (TUNEL), was unchanged at this early time point in response to the decrease in Myo/Nog cells. However, increasing the number of Myo/Nog cells within the lesion by injecting BAI1-positive (+) cells isolated from the brains of other animals, significantly reduced cell death and increased the number of NeuN+ neurons compared to brains injected with phosphate buffered saline or exogenous BAI1-negative cells. These findings demonstrate that Myo/Nog cells rapidly react to injury within the brain and increasing their number within the lesion is neuroprotective.

## Introduction

Microhemorrhages contribute to the pathogenesis of central nervous system disorders, including Alzheimer’s disease and cerebral amyloid angiopathy (Cullen et al., 2005, 2006; Stone, 2008; Stone et al., 2015). Animal models that induce microhemorrhages are useful for defining the cellular responses to injury and testing approaches to reduce neuronal damage. One such model, developed by Purushothuman et al. (2013), involves insertion of a needle into the posterior parietal cortex and hippocampus of the rat brain. The needlestick (NS) injury results in hemorrhage, cell death, and formation of extracellular deposits of amyloid beta (Aβ) and tau along the lesion. The area flanking the lesion exhibits oxidative stress, increased microglia, astrogliosis and increased expression of Aβ, amyloid precursor protein, hyperphosphorylated tau, fibroblast growth factor 2, and neuroglobin (Purushothuman et al., 2013; Purushothuman and Stone, 2015).

In our studies of the retina, we identified a subpopulation of cells, called Myo/Nog cells, with neuroprotective properties (Bravo-Nuevo et al., 2016; Brandli et al., 2017). Myo/Nog cells were first discovered in the early chick embryo by their expression of the bone morphogenetic protein (BMP) inhibitor Noggin (Gerhart et al., 2000, 2004, 2006), the skeletal muscle specific transcription factor MyoD and brain-specific angiogenesis inhibitor 1 (BAI1) (Gerhart et al., 2020a). Release of Noggin from Myo/Nog cells restricts the field of BMP signaling and is essential for normal development of the central nervous system, eyes and skeletal, and cardiac muscle (Gerhart et al., 2006, 2009). Anencephaly or microcephaly are common occurrences in embryos in which Myo/Nog cells are depleted in the epiblast before the onset of gastrulation (Gerhart et al., 2011). Malformations are largely prevented with their reintroduction into the blastocyst or addition of Noggin-soaked beads into the mesoderm (Gerhart et al., 2006, 2009, 2011).

Neonatal and adult tissues also contain Myo/Nog cells where they are present in low numbers until activated by injury (Walker et al., 2010; Gerhart et al., 2012, 2014, 2018, 2019b, 2020c; Brandli et al., 2017). They are the source of contractile myofibroblasts in the lens and synthesize skeletal muscle proteins in human preretinal membranes (Gerhart et al., 2014, 2017, 2019b, 2020c). Myo/Nog cells lack detectable levels of NeuN, glial fibrillary acid protein and markers of microglia in the mouse brain and retinas of mice and rats (Bravo-Nuevo et al., 2016; Brandli et al., 2017; Gerhart et al., 2020a), although like microglia, they engage in phagocytosis (Gerhart et al., 2020b). Oxygen-induced retinopathy in mice is accompanied by an expansion of the population of Myo/Nog cells and their homing to areas of stress and cell death. Their depletion in the hypoxic retina results in an increase in neuronal cell death (Bravo-Nuevo et al., 2016). Similar behaviors of Myo/Nog cells were observed in the light damaged retina of albino rats in which intravitreal injection of exogenous Myo/Nog cells reduced cell death, improved visual function and dampened the Muller cell stress response (Brandli et al., 2017). In this study, we examined the acute response of Myo/Nog cells to NS injury and the effects of decreasing and increasing the population on cell death.

## Materials and Methods

### Animals

Male and female albino Sprague-Dawley rats, ages 3–6 months, were obtained from Charles River Laboratories (Wilmington, MA, United States) and the Animal Resource Centre (Perth, WA, Australia). Rats were raised in cycles of 12 h of light and 12 h of darkness. All experimental procedures were approved by the Philadelphia College of Osteopathic Medicine’s Institutional Animal Care and Use Committee (IACUC) and the University of Sydney’s Animal Ethics Committee.

### Needlestick Injury

Needlestick injury was performed as described previously (Purushothuman et al., 2013). Briefly, a 1.5-mm hole was drilled through the skull bilaterally using a burr hole drill approximately 3.5 mm caudally from bregma and 2 mm laterally from the midline. A 0.64-mm (23-gauge) needle was inserted through the burr hole to a depth of 5 mm inferior to the dura mater to penetrate the posterior parietal cortex and hippocampus and remained there for 5 s before being removed. Concurrent with the removal of the needle, the rats were injected with phosphate buffered saline (PBS) or cells in PBS as described below. The rats were sacrificed 24 h after NS injury and perfused transcardially with 4% paraformaldehyde.

### Myo/Nog Cell Depletion

The anti-BAI1 G8 IgM monoclonal antibody (mAb) was generated by immunizing mice with chick embryo paraxial mesoderm cells (Gerhart et al., 2001). The G8 mAb specifically binds to cells that express both Noggin and MyoD in multiple species (Gerhart et al., 2001). Lysis of Myo/Nog cells was carried out by injecting 1 μl of a pre-mixed solution of the G8 mAb diluted 1:20 and anti-mouse complement (0.25 mg) (Rockland, Limerick, PA, United States) into the NS lesion at the time of withdrawal of the needle (Gerhart et al., 2006).

### Myo/Nog Cell Extraction

Isolation of Myo/Nog cells from the rat brain was performed by magnetic cell sorting using the anti-BAI1 G8 mAb as previously reported (Brandli et al., 2017). Briefly, the brain was sliced into small pieces and incubated in 0.25% Trypsin-EDTA (Thermo Fisher Scientific, Waltham, MA, United States) at 37°C for 15 min. A single cell suspension was produced by trituration. The population that non-specifically bound magnetic anti-mouse IgM secondary antibodies was removed on the cell sorting column (Miltenyi Biotec, Auburn, CA, United States). Remaining cells were incubated with the anti-BAI1 mAb and magnetic anti-IgM secondary antibodies (Miltenyi Biotec). The cells that did not bind to the column were considered BAI1-negative (−). Bound, BAI1-positive (+) cells were eluted from the column in buffer according to the manufacturer’s instructions.

### Treatment Groups

The six treatment groups included: (1) uninjured control; (2) NS with injection of 5 μl of PBS; (3) NS with injection of complement in 5 μl of PBS; (4) NS with injection of BAI1 mAb and complement in 5 μl of PBS; (5) NS with injection of 25,000 BAI1+ brain cells in 5 μl of PBS; and (6) NS with injection of 25,000 BAI1− brain cells in 5 μl of PBS. Both hemispheres of 3–6 animals per experimental group were treated.

### Immunohistochemistry and Analysis

Fixed tissue was immersed in 30% sucrose for a minimum of 24 h. Coronal sections of approximately 1 mm were cut with a razor blade around the area of the NS injury. The tissue was placed into a cassette containing Tissue-Tek Optimum Cutting Temperature (OCT) gel (Thermo Fisher), frozen on dry ice and stored at −80°C. Tissue was sectioned at 20 μm using a Leica CM cryostat (Leica Biosystems, Buffalo Grove, IL, United States).

Sections were double labeled with the BAI1 mAb and a goat anti-mouse polyclonal antiserum to Noggin diluted 1:200 (R&D Systems, Minneapolis, MN, United States). Single labeling was carried out with terminal deoxynucleotidyl transferase-mediated dUTP-biotin nick end labeling (TUNEL) reagents (Roche Applied Science, Penzberg, Germany) or a rabbit mAb to NeuN (Abcam, Cambridge, MA, United States), a marker for mature neurons. Secondary antibodies included goat anti-rabbit IgG Alexa Fluor 488, rabbit anti-goat IgG 488 and goat anti-mouse IgM μ chain, goat-anti-mouse IgG and donkey anti-goat IgG conjugated with DyLights 488 or 549 (Jackson ImmunoResearch, West Grove, PA, United States). Nuclei were stained with 4′,6-diamidino-2-phenylindole (DAPI) contained in the Shield Mounting Medium (Electron Microscope Sciences, Hatfield, PA, United States).

Four to eight sections of each rat hemibrain (8–16 sections/brain) were scored for single and double labeled cells in each experimental group and immunofluorescence localization protocol. Labeled cells were counted within 1 mm of the NS track using a Nikon Eclipse E800 epifluorescence microscope equipped with the Evolution QE Optronics video camera and Image Pro Plus image analysis software program (Media Cybernetics, Rockville, MD, United States). Photographs were adjusted for brightness and contrast with Adobe Photoshop 2021 (Adobe Systems Inc., San Jose, CA, United States). Images also were acquired with an Olympus Fluoview FV1000 confocal microscope. Occasionally, minor changes in gain or offset were needed to retain optimal intensity range, as determined by the confocal software saturation and black level detectors. Settings were changed equally across conditions and channels during imaging.

### Statistical Analyses

Statistical analyses were performed using 2019 Minitab, LLC (State College, PA, United States). A Grubbs’ outlier test was performed to calculate outliers in the data. Levene’s method for variances was used to determine equal or unequal variance in the cell counts. One-way ANOVA was computed to determine statistical significance within the treatment groups. An α = 0.05 and 95% confidence interval was used for all calculations of significance. Significant differences between pairs of treatment groups (*p*-values) were obtained with the Games Howell *post hoc* test for unequal variances [Myo/Nog and TUNEL-positive (+) cells] and Tukey *post hoc* for equal variances (NeuN+ cells).

## Results

### Response of Myo/Nog Cells Within 24 h of Needlestick Injury

**FIGURE 1:**
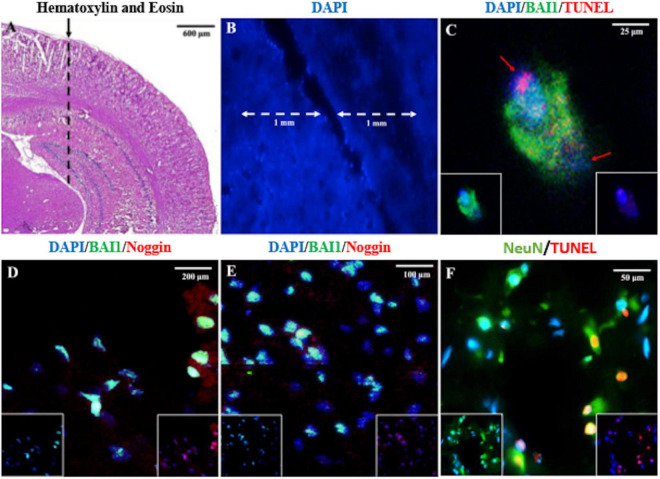
Identification of the NS injury tract lesion site, Myo/Nog cells and dying neurons in the rat brain. The area where the needle is inserted into parietal cortex and hippocampus is shown in the hematoxylin and eosin stained section in **(A)** (dashed line). The NS tract and the counting area within 1 mm of the lesion is shown in **(B)**. Sections were double labeled with antibodies to BAI1 (green) and TUNEL reagents (red), BAI1 (green), and Noggin (red) **(D,E)**, and NeuN (green) **(F)** and TUNEL (red) **(C)**. Nuclei were stained with DAPI in **(B–F)**. Overlap of green and red appear yellow in merged images. Unmerged images are shown as insets at the bottom of the photographs in **(C–E)**. Images were acquired from the uninjured posterior parietal cortex and hippocampus **(A)**, along the NS tract within the parietal cortex **(B,D)**, interface of the parietal cortex and hippocampus **(C,F)**, and the end of the NS tract in the hippocampus **(E)**. A BAI1+ Myo/Nog cell appears to have phagocytosed a TUNEL+ cell **(C)**. The red arrows in **(C)** depict separate nuclei. Myo/Nog cells co-expressed BAI1 and Noggin **(D,E)**. The majority of TUNEL+ cells were NeuN+ neurons **(F)**.

A NS penetrating the posterior parietal cortex and hippocampus (Figures 1A,B) was used to examine the acute behavior of Myo/Nog cells in response to focal injury of the rat brain. Analyses were carried out 24 h post-injury to coincide with the previously reported peak in cell death (Purushothuman et al., 2013). Myo/Nog cells were identified by double labeling with antibodies against BAI1 and Noggin. In the uninjured and injured brain, BAI1 co-localized with Noggin (Figures 1D,E). Single labeled cells were rarely observed throughout the tissue.

**FIGURE 2:**
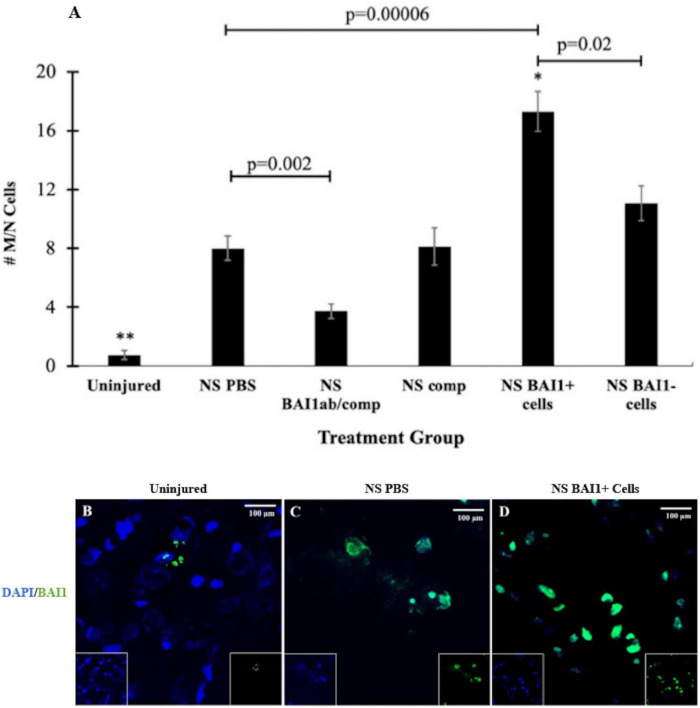
Comparison of the number of Myo/Nog cells before and after NS injury and with depletion or addition of BAI1+ cells. Six tissue sections from 3 to 6 animals per treatment group were labeled with the BAI1 mAb (green in **B–D**). Nuclei were stained with DAPI (blue in **B–D**). BAI1+ cells were counted within a 1 mm area lateral to NS tract or in a similar area in the uninjured brain. The results are the means ± SEM 24 h after NS with injection of PBS, the BAI1 mAb and complement (BAI1ab/comp), complement alone (comp), BAI1+ cells or BAI1– cells **(A)**. NS injury significantly increased the number of Myo/Nog cells compared to a similar region in the uninjured brain (^**^p = 0.00001). Treatment with the BAI1 mAb and complement (abBAI1/comp) significantly reduced the number of Myo/Nog cells compared to NS with PBS (*p* = 0.002), BAI1+ cells (*p* = 0.00001), BAI1– cells (*p* = 0.0002), or comp alone (*p* = 0.04). Significantly more Myo/Nog cells were present in brains injected with Myo/Nog cells than the other groups (*p < 0.05). Myo/Nog cells were most prevalent after injection of exogenous BAI1+ cells (**D**; *p* = 0.00006). Unmerged images are shown as insets at the bottom of the photographs of merged images. Panels **(B–D)** are photographs of BAI1 and DAPI labeling in the parietal cortex of uninjured brain and NS injured brains injected with PBS or BAI1+ cells. More Myo/Nog cells were observed after injecting BAI1+ cells than in uninjured brains and those injected with PBS.

Myo/Nog cells were present in low numbers, either as single cells or in small clusters, in the uninjured rat brain (Figures 1D, 2A,B) and did not appear to be concentrated in any area of the brain. The population increased eightfold in a 1 mm area around the lesion within 24 h of the NS (Figures 2A,C). Expansion of the Myo/Nog population was observed in both the posterior parietal cortex (Figures 1D, 2C) and hippocampus (Figure 1E), near the NS injury injection site. These results demonstrate that Myo/Nog cells are the primary source of Noggin and expressors of BAI1 before and after focal brain injury, and they rapidly increase in response to focal brain injury along the lesion.

Occasionally, two nuclei of different sizes were observed at different poles within BAI1+ cells (Figure 1C). In the example shown in Figure 1C, BAI1 staining appeared to be surrounding the TUNEL+ nucleus. This suggests that Myo/Nog cells may be engulfing apoptotic cells, as observed previously in the skin and eyes (Gerhart et al., 2020b).

### Effects of Reducing the Population of Myo/Nog Cells Along the Needlestick Track on Cell Death and Neurons

**FIGURE 3:**
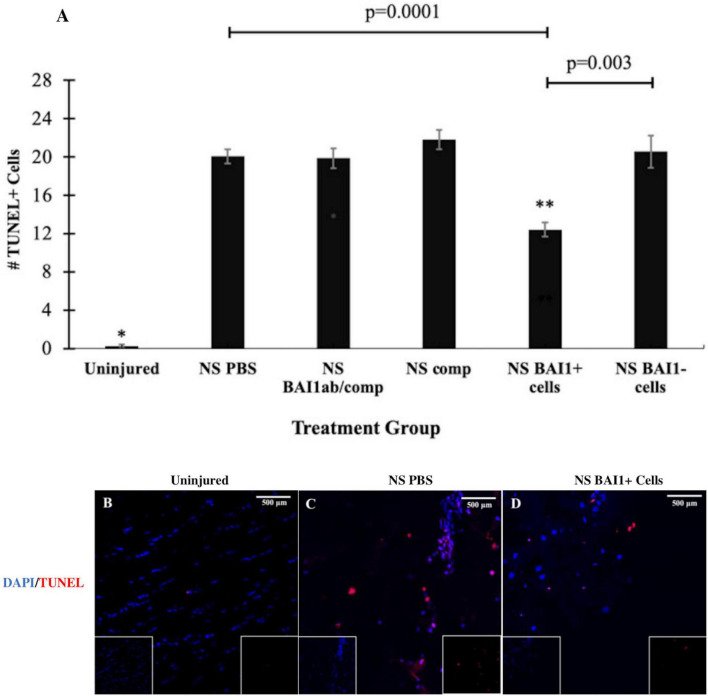
Comparison of cell death before and after NS injury and with depletion or addition of BAI1+ cells. Six tissue sections from 3 to 6 animals per treatment group were stained with TUNEL reagents (red in **B–D**). Nuclei were stained with DAPI (blue in **B–D**). The number of TUNEL+ cells was counted within a 1 mm area lateral to NS tract or in a similar area in the uninjured brain. The results are the means ± SEM 24 h after NS with injection of PBS, the BAI1 mAb and complement (BAI1ab/comp), complement alone (comp), BAI1+ cells or BAI1– cells **(A)**. Significantly fewer TUNEL+ cells were present in uninjured tissue than the other treatment groups (*p = 0.00001). NS injury significantly increased the number of TUNEL+ cells in all treatment groups (*p* < 0.05). Injection of BAI1+ cells isolated from the brains of other animals significantly reduced the number of TUNEL+ cells compared to all other groups (^**^p < 0.05). The numbers of TUNEL+ cells were similar in brains injected with PBS, BAI1 and complement (BAI1Ab/comp), and complement alone (Comp). Panels **(B–D)** are photographs of TUNEL and DAPI labeling in the parietal cortex of the uninjured brain and NS injured brains injected with PBS or BAI1+ cells. Unmerged images are shown as insets at the bottom of the photographs of merged images. TUNEL+ cells were rare in the uninjured brain **(B)**. Fewer TUNEL+ cells were observed after injecting BAI1+ cells **(D)** than in those injected with PBS **(C)**.

Consistent with previous results (Purushothuman et al., 2013), NS injury caused an increase in cell death along the lesion as measured by TUNEL staining (Figures 1F, 3A–C). The vast majority of the TUNEL-positive (+) cells were labeled with the NeuN antibody (Figure 1F). The BAI1 mAb was injected with complement to examine whether reducing the population of Myo/Nog cells would affect cell viability. This method of targeted, complement mediated cell lysis decreased the number of Myo/Nog cells in a 1 mm area surrounding the lesion by approximately 50% compared to NS with an injection of PBS or complement alone (Figure 2A). Injection of only complement had no effect on the size of the Myo/Nog population (Figure 2A).

**FIGURE 4:**
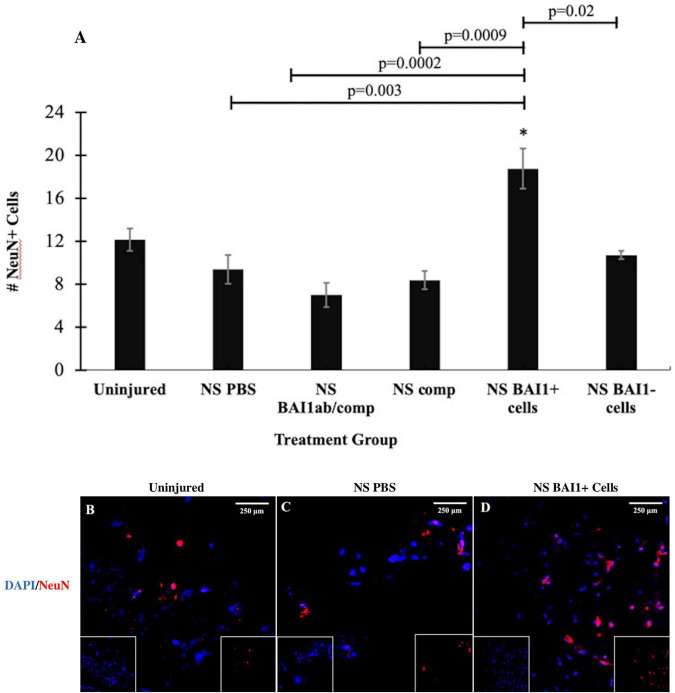
Comparison of the number of NeuN+ neurons before and after NS injury and with depletion or addition of BAI1+ cells. Six tissue sections from 3 to 6 animals per treatment group were stained with TUNEL reagents (red in **B–D**). Nuclei were stained with DAPI (blue in **B–D**). NeuN positive cells were counted within a 1 mm area lateral to NS tract or in a similar area in the uninjured brain. The results are the means ± SEM 24 h after NS with injection of PBS, the BAI1 mAb and complement (BAI1ab/comp), complement alone (comp), BAI1+ cells or BAI1– cells **(A)**. The elevation in the number of NeuN cells in brains injected with BAI1+ compared to the uninjured brain was not statistically significant (*p* = 0.06). The number of NeuN+ cells was significantly greater after injection of BAI1+ cells than in the other treatment groups (*p ≤ 0.05). Panels **(B–D)** are photographs of NeuN and DAPI Unmerged images are shown as insets at the bottom of the photographs of merged images.

Reducing the number of Myo/Nog cells with the BAI1 mAb and complement did not alter the number of TUNEL+ cells within the 24-h period following injury (Figure 3A). The effect of Myo/Nog cell reduction on the population of neurons was determined by labeling with an antibody to NeuN. A small but statistically insignificant reduction in the number of NeuN+ neurons was observed along the NS track injected with PBS (Figure 4A). Targeted depletion of Myo/Nog cells further reduced the number of neurons, but the difference between treatment with the BAI1 mAb and complement versus NS with PBS were not significant (Figure 4A).

### Effects of Adding Myo/Nog Cells Along the Needlestick Track on Cell Death and Neurons

Myo/Nog cells were added to the lesion by injecting BAI1+ cells isolated from the brains of other rats as the needle was withdrawn from the brain. The number of Myo/Nog cells was significantly elevated 24 h after their injection compared to the uninjured control group and those that received the NS and PBS (Figures 2A–D). Addition of BAI1− cells obtained from the cell sort resulted in a small increase in the endogenous population of Myo/Nog cells compared to NS with PBS but the difference was not statistically significant (Figure 2A).

The effect of exogenous Myo/Nog cells on cell death was quantified within 1 mm of the NS track by TUNEL labeling 24 h after injury. Addition of brain-derived BAI1+ cells significantly reduced the number of TUNEL+ cells compared to brains injected with PBS or BAI1− cells (Figures 3A,C,D). Exogenous BAI1− cells had no effect on cell death (Figure 3A). The number of NeuN+ neurons cells within 1 mm of the wound was significantly increased with the addition of BAI1+ cells compared to brains injected with PBS or BAI1− cells (Figures 4A,C,D). Injection of BAI1+ cells also increased the number of NeuN+ cells compared to uninjured brains, but the effect was not statistically significant (Figures 4A,B,D).

## Discussion

Myo/Nog cells are normally present in low numbers in all embryonic and adult tissues analyzed thus far (Gerhart et al., 2004, 2006, 2009, 2011, 2012; Bravo-Nuevo et al., 2016; Brandli et al., 2017). A variety of stimuli activate, induce proliferation of and recruit Myo/Nog cells, including cell death, epidermal abrasion, tumor formation, surgical wounding of the lens, and retinopathy induced by hypoxia and light damage (Brandli et al., 2017; Gerhart et al., 2014, 2019a, 2020b). In this study, we examined the response of Myo/Nog cells to a focal injury within the brain. As in the mouse brain (Gerhart et al., 2020a), they constituted a minor population in the uninjured neocortex and hippocampus. Within 24 h of NS injury, the number of Myo/Nog cells increased eightfold adjacent to the NS track. The source of Myo/Nog cells in the lesion is unknown. At least some of these cells were likely to have arisen from the proliferation and migration of resident Myo/Nog cells as observed in other tissues. In the skin, Myo/Nog cells are present in a niche associated with the hair follicles (Gerhart et al., 2012). The population rapidly expands and enters the surrounding dermis in response to abrasion, suggesting that Myo/Nog cells had migrated from the niche into the wound (Walker et al., 2010; Gerhart et al., 2012). The increase in Myo/Nog cells in the injured lens results from activation of the endogenous population; however, an additional source of these cells is the ciliary body *via* migration on the zonules of Zinn (Gerhart et al., 2018). Analyses of cell proliferation in response to brain injury will shed light on the contribution of locally derived Myo/Nog cells to the wound versus possible recruitment from the vasculature.

The impact of the expanded population of endogenous Myo/Nog cells along the lesion is also a subject for further study. The NS itself caused an elevation in TUNEL staining within 24 h with a small, but statistically insignificant, loss of NeuN+ cells, suggesting that dying and dead neurons had not yet been cleared. Depletion of approximately 50% of the Myo/Nog population along the NS track did not significantly alter the number of TUNEL+ or NeuN+ cells, although it is possible that their complete elimination in the area of injury or analyses at later time points would reveal an effect on cell viability.

The impact of further increasing the Myo/Nog population along the lesion was tested by adding BAI1+ cells isolated from the brains of other rats. This procedure produced an increase in the number of BAI1+/Noggin+ within 1 mm around the lesion, and in the hippocampus. The elevation in Myo/Nog cells significantly reduced cell death and increased the number of NeuN+ neurons along the lesion compared to injection of PBS or BAI1− cells. The elevation in Myo/Nog cells in response to injection of BAI1− cells, although not statistically significant, could reflect a response of the endogenous population to exogenous cells or the molecules they release.

Myo/Nog cells are the primary source of Noggin in the rat brain, as they are in other tissues (Gerhart et al., 2006, 2009, 2011, 2012, 2014, 2020a). It is reasonable to hypothesize that Noggin produced by the exogenous Myo/Nog cells is at least partially responsible for the reduction in cell death and increase in neurons following NS injury. In support of this hypothesis, overexpression or infusion of Noggin reduces neuronal cell death and facilitates neuronal repair in several models of neuronal injury (Samanta et al., 2010; Dizon et al., 2011; Shin et al., 2014, 2018; Morell et al., 2015; Lu et al., 2016; Zhu et al., 2017; Diaz-Moreno et al., 2018; Harnisch et al., 2019).

Noggin also regulates neurogenesis and stem cell behavior in the adult brain. Expression of Noggin and BMPs were mapped to the subgranular zone (SGZ) of the dentate gyrus of the hippocampus (Valenzuela et al., 1995; Lim et al., 2000; Peretto et al., 2004; Colak et al., 2008; Morrell et al., 2016). This region, along with the walls of the lateral ventricles, are sites of neurogenesis in the adult brain (Gage, 2010; Morrell et al., 2016). Alteration of BMP signaling affects the size of the stem and progenitor cell populations (Bonaguidi et al., 2005, 2008). Targeted expression of Noggin in stem cells in the SGZ promotes expansion of progenitor populations and a shift in fate from astrocyte differentiation to the neuronal and oligodendrocyte lineages (Morrell, 2010). A rapid stimulation of stem and progenitor cell proliferation in response to the NS, along with an elevation in Noggin released from Myo/Nog cells, could explain the increase in NeuN+ cells seen in the hippocampus after their addition to the lesion compared to uninjured tissue or injection of BAI1− cells or PBS. This is an important area for exploration in the context of Myo/Nog cells’ therapeutic potential.

Another effect of Noggin overexpression in the ischemic model of brain injury is an increase in microglia (Samanta et al., 2010; Shin et al., 2012, 2014). Infusion of Noggin decreases pro-inflammatory M1 macrophages and increases anti-inflammatory M2 macrophages that facilitate wound repair and remodeling (Shin et al., 2014; Zhao et al., 2002, 2003). Ischemic brain injury models revealed that astrocytes and microglia exhibit a gliotic reaction in response to BMPs, and Noggin circumvents gliosis (Peretto et al., 2004; Colak et al., 2008; Gage, 2010; Morrell et al., 2016). NS injury also promotes proliferation of microglia and astrogliosis (Purushothuman et al., 2013). In the retina, injection of BMP7 triggers reactive gliosis with increased expression of glial fibrillary acidic protein (GFAP) and Muller cell hypertrophy (Dharmarajan et al., 2014). Our study of the light damaged retina revealed that intravitreal injection of brain-derived Myo/Nog cells reduces Muller cell length and their expression of GFAP, in addition to decreasing cell death and improving vision (Brandli et al., 2017). These studies suggest that regulation of BMP signaling *via* the release of Noggin from Myo/Nog cells may be neuroprotective in the retina, in part by reducing gliosis. Examination of glial cell activation in response to elevated Myo/Nog cells in the NS model of focal brain injury may reveal mechanisms of action common to both tissues.

Myo/Nog cells differentiate into myofibroblasts in the lens and they synthesize skeletal muscle proteins in human preretinal membranes (Gerhart et al., 2014, 2017, 2019b, 2020c). Vanlandewijck et al. (2018) surveyed the whole brain and found two types of fibroblast-like cells. Although the markers used by that group have not been examined for expression in Myo/Nog cells; however, it is possible that Myo/Nog cells could be the source of some of those fibroblasts.

Myo/Nog cells are distinct from microglia in the brain and retina, and macrophages outside of the central nervous system (Gerhart et al., 2012, 2020a, b; Bravo-Nuevo et al., 2016), still, they do engage in phagocytosis (Gerhart et al., 2020b). Myo/Nog cells internalize tattoo ink in the skin, beads injected into the aqueous humor and dead cells in the injured lens (Gerhart et al., 2020b). BAI1 is a receptor for phosphatidylserine present in the outer leaflet of the plasma membrane of dying cells with binding triggering engulfment (Park et al., 2007; Hochreiter-Hufford et al., 2013; Penberthy and Ravichandran, 2016). Thus, Myo/Nog cells may be homing to the area of cell death along the NS track and participating in clearance, as suggested by the presence of BAI1+ cells with two nuclei, one of which was TUNEL+.

## Conclusion

In summary, Myo/Nog cells are the primary source of Noggin and BAI1 within the uninjured and injured posterior parietal cortex and hippocampus of the rat. They react to focal injury by expanding in number around the lesion and may engage in clearance of dying cells. Supplementation with exogenous Myo/Nog cells reduces cell death and increases the number of neurons adjacent to the NS tract. The long-term impact of an increase or decrease in Myo/Nog cells, along with defining the molecular mechanisms regulating their homing to the wound and mediation of neuroprotection are areas for further study.

## Data Availability Statement

The original contributions presented in the study are included in the article/supplementary material, further inquiries can be directed to the corresponding author.

## Ethics Statement

The animal study was reviewed and approved by the Philadelphia College of Osteopathic Medicine Institutional Animal Care and Use Committee and the University of Sydney’s Animal Ethics Committee.

## Author Contributions

AB-N, JS, and SP contributed to conceptualization of the study. AB-N, MG-W, SP, SJ-P, MB, NM, AP, PL, JK, MM, JM, LG, and E-JT performed the data curation. AB-N, MG-W, NM, AP, and SJ-P completed the formal analysis. AB-N, JS, SJ-P, SP, JG, MG-W, and GG developed a laboratory methodology. AB-N, MG-W, and JS performed the project administration. AB-N, MG-W, JS, and JG provided the resources for the study. AB-N, MG-W, and NM completed the validation of the study and data. AB-N, JG, NM, and MM acquired and created the visualization and imaging. AB-N wrote the first draft of the manuscript. All authors contributed to manuscript revision, read, and approved the submitted version.

## Conflict of Interest

The authors declare that the research was conducted in the absence of any commercial or financial relationships that could be construed as a potential conflict of interest.

## Publisher’s Note

All claims expressed in this article are solely those of the authors and do not necessarily represent those of their affiliated organizations, or those of the publisher, the editors and the reviewers. Any product that may be evaluated in this article, or claim that may be made by its manufacturer, is not guaranteed or endorsed by the publisher.
